# Segmentation of Drug-Treated Cell Image and Mitochondrial-Oxidative Stress Using Deep Convolutional Neural Network

**DOI:** 10.1155/2022/5641727

**Published:** 2022-05-26

**Authors:** Awais Khan Nawabi, Sheng Jinfang, Rashid Abbasi, Muhammad Shahid Iqbal, Md Belal Bin Heyat, Faijan Akhtar, Kaishun Wu, Baidenger Agyekum Twumasi

**Affiliations:** ^1^School of Computer Science and Engineering, University of Central South University, Hunan, China; ^2^School of Information and Communication Engineering, University of Electronics Science and Technology, Chengdu, China; ^3^Anhui Polytechnic University, Wuhu, Anhui, China; ^4^School of Computer Science and Technology, Anhui University, Hefei, China; ^5^Department of Computer Science, Air University, Islamabad, Pakistan; ^6^IoT Research Center, College of Computer Science and Software Engineering, Shenzhen University, Shenzhen, Guangdong 518060, China; ^7^School of Computer Science and Engineering, University of Electronic Science and Technology of China, Chengdu, Sichuan, China; ^8^Department of Electrical and Electronic Engineering, Ho Technical University, Ho, Ghana

## Abstract

Most multicellular organisms require apoptosis, or programmed cell death, to function properly and survive. On the other hand, morphological and biochemical characteristics of apoptosis have remained remarkably consistent throughout evolution. Apoptosis is thought to have at least three functionally distinct phases: induction, effector, and execution. Recent studies have revealed that reactive oxygen species (ROS) and the oxidative stress could play an essential role in apoptosis. Advanced microscopic imaging techniques allow biologists to acquire an extensive amount of cell images within a matter of minutes which rule out the manual analysis of image data acquisition. The segmentation of cell images is often considered the cornerstone and central problem for image analysis. Currently, the issue of segmentation of mitochondrial cell images via deep learning receives increasing attention. The manual labeling of cell images is time-consuming and challenging to train a pro. As a courtesy method, mitochondrial cell imaging (MCI) is proposed to identify the normal, drug-treated, and diseased cells. Furthermore, cell movement (fission and fusion) is measured to evaluate disease risk. The newly proposed drug-treated, normal, and diseased image segmentation (DNDIS) algorithm can quickly segment mitochondrial cell images without supervision and further segment the highly drug-treated cells in the picture, i.e., normal, diseased, and drug-treated cells. The proposed method is based on the ResNet-50 deep learning algorithm. The dataset consists of 414 images mainly categorised into different sets (drug, diseased, and normal) used microscopically. The proposed automated segmentation method has outperformed and secured high precision (90%, 92%, and 94%); moreover, it also achieves proper training. This study will benefit medicines and diseased cell measurements in medical tests and clinical practices.

## 1. Introduction

**Figure 1:**
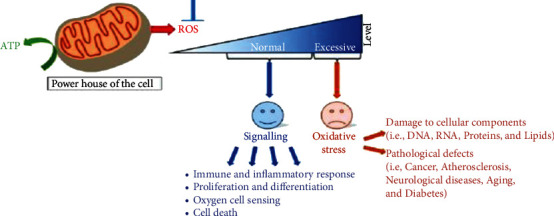
Oxidative stress: mitochondrial cell, powerhouse, normal and excessive level, oxidative stress, and damage to cellular components; through image segmentation, mitochondrial cell image segmentation plays a vital role for stress; dataset was collected of mitochondria cell images; and apply deep learning to identify the segment [34].

Mitochondria are powerhouses of cells that provide energy for various functions. Oxidative stress means that during multiple processes in mitochondria, reactive oxygen species (ROS) are produced, which cause DNA/RNA mutations, Alzheimer's disease [1–3], ageing, and cell death, as shown in Figure 1. Image segmentation, a renowned term used in medical imaging, refers to the partitioning of digital cell images into multiple subsegments intended to analyse an idea to get something meaningful. In biological sciences, large amount of prints are produced microscopically, where image segmentation plays a significant role in extracting meaningful information from extensive image data. On the other hand, drugs are used extensively in the clinic and help produce new compounds and investigate the effects on cells [4–6]. To examine the results of the mixture on cells, bulk of cell images is generated by advanced microscopes during experiments which persuade analysts toward image analysis. Image segmentation is one of the critical aspects of image analysis [7–9]. Cell culture and drug process and convolutional neural network (CNN) [10, 11] have been applied to cell biology. However, as there is a great demand to train a large number of reliable training data, it is a vital problem; it is critical to analyze drugs and pathological cells to obtain high-quality labeled images. A well-segmented cell image can help to quickly and accurately label the image. For further phenotypical analysis, some cell images are extracted. In the literature, different image segmentation algorithms have been proposed. Among those, a critical and experimental way is extracting image features by using statistics. Statistical methods are characterized by image modelling. Every pixel in the image is considered the variable's probability distribution, where the probability of a pixel combination is the greatest from a statistical point of view.

Previous studies, including three-dimensional morphological operations, threshold, statistical model, deformable model, and image segmentation methods, have been applied to magnetic resonance image segmentation [12–14]. These methods of segmentation are based on a complex algorithm. The accuracy of the deformation model is 1.44 + 1.1 for the hip magnetic resonance imaging dataset. The statistical model archiving accuracy was 1.21 + 0.53 mm. These methods have achieved reasonable femoral segmentation on the MR image set. They have some limitations (making time necessary for proximal femoral segmentation and changing the height of the femoral shape). CNNs recognize images, process natural languages, and recognize speech [15–18]. In recent years, in-depth learning in medical imaging, especially in computer-aided diagnostics and image segmentation, has been successful. In the past, the manual function was used in the MR image set, and the neural network automatically learns complex functions from data. In the first application of CNN in medical image segmentation, the pyramid convolutional neural network architecture was used [19]. The pyramid CNN structure segmented the proximal femur to achieve a moderate segmentation effect [20]. The development of image segmentation technology based on a complete network structure led to more accurate pixel segmentation. These networks use an encoder-decoder type architecture in which the decoder network functions project low-resolution encoder mapping features to high-resolution pixel classification features [21]. CNN architecture based on encoder decoders has recently been widely used in biomedicine, providing accurate image segmentation. For example, the two-dimensional encoder-decoder network structure and the 3-dimensional connection component analysis or the 3-dimensional simple deformation models provide the final 3-dimensional segmentation mask [22]. In addition, a cascaded two-dimensional neural network with an intermediate statistical model for the segmentation of the knee meniscus is proposed, which is used to generate smaller patch input for the three-dimensional neural network model. The author introduces cytological analysis computational tools such as cell segmentation deep learning techniques capable of processing both free-floating and clumps of abnormal cells from digitised images of traditional Pap smears with a high overlapping rate, and cell image segmentation, in previous studies, no one proposed image segmentation for drug, diseased, and mitochondria cell, and some authors proposed image segmentation for medical, but no one proposed image segmentation for drug-treated image cell, diseased cell image, and mitochondria cell image, there is a gap in this area, that is why we work in this area, and we proposed a new algorithm for image segmentation, drug, diseased cell image, and mitochondria cell image [23, 24]. More research is needed in this area, particularly in mitochondrial cells for measuring oxidative stress using machine learning.

In this paper, we propose investigating convolutional neural network architecture based on drug-treated images and comparing its automatic segmentation performance with various methods for drug treatment [25–28] and disease affected by the image, as well as the reference standard of expert manual segmentation [29, 30]. We experimented with three different convolutional neural network architectures: multiple initial feature maps, layers, and scalability training. Using quadruple cross-validation, we tested their segmentation performance and the golden standard of manual segmentation. Different convolutional neural network architectures are implemented by changing the number of feature graphs and the coding and decoding layer. The influence of architecture design parameters on segmentation performance is analyzed [31]. Furthermore, we extended the convolutional neural network architecture to connect the extended convolutions with different spreading rates at the encoder-decoder architecture's central layer. According to theories, one of the causes of ageing is cumulative damage to mitochondria and mitochondrial DNA (mtDNA) caused by reactive oxygen species (ROS) [32]. While stress can be an oxidative challenge for creatures, defensive mechanisms such as overregulation of antioxidant defences and decreased mitochondrial effectiveness appear to do so in king penguins, allowing them to cope with their changing and soothing environment [33]. In this study,

1. we proposed a deep learning method for image segmentation, mitochondrial, drug-treated, and diseased image segmentation (DNDIS)
2. our method can quickly segment mitochondrial cell images without supervision
3. our method can further segment the highly drug-treated, diseased and mitochondrial cell images
4. we used deep learning, Resnet-50, and convolutional neural network (CNN)
5. we input cell images and apply building block and used ResNet-50 to detect the mitochondrial, drug-treated, and diseased part from cell image
6. the red box is the last detection image mark, false positive, and genuinely positive and, finally, the segmentation of the highly treated drug image part
7. we segmented the cell position and detect the heterogeneity between cells
8. mitochondrial cell image segmentation plays a vital role for stress

## 2. Related Work

Volumetric computer division is a subfield of computer science. Connectomics, the study of brain wiring outlines, has primarily determined mitochondrial cell information, with CNNs for cell limit division being proposed early [35, 36]. Furthermore, fruitful methodologies for the division of neurotransmitters, a task similar to mitochondrial division, have been proposed [37, 38].

Several strategies for programmed mitochondrial division have been proposed based on previous research. Liu et al. [39] suggested using the mask R-CNN [40] to separate SEM images. Their primary focus is on postprocessing division covers obtained through the extensive organisation. The postprocessing is performed in three stages: first, a morphological opening activity is used to remove small areas and large smooth areas; second, a multifacet (3D) data combination calculation is used to remove mitochondria that are more limited than a set edge; and finally, a multifacet (3D) data combination calculation is used to remove mitochondria that are more limited than a set edge. Finally, an analysis is used to work on the consistency in the adjacent variables. Oztel et al. [41] also proposed combining a deep CNN with postprocessing. They created their own CNN design in which preparation is completed by removing 32 × 321 × 1 noncovering blocks from the preparation volume in electron microscopy volumes. A common name is assigned to each block based on the number of pixels from the mitochondria and nonmitochondrial classes. The organization's final completely associated layer generates two-channel mitochondria versus nonmitochondrial class scores, converted to double grouping. They also present three postprocessing stages: 2D fake discovery sifting, limit refinement, and 3D separating. Each of the proposed methodologies produces promising results, but, unlike our strategy, they do not use 3D spatial data in network planning.

While all presented methodologies use 2D convolutions, Haberl et al. [42] introduced the CDeep3M, a 3D convolution-based approach. A ready-to-use volumetric division arrangement uses DeepEM3D, a cloud-based profound CNN [43]. The results of mitochondrial division with DeepEM3D do not outperform state-of-the-art results. Still, the methodology is intriguing because it is exceptionally robust and achieves excellent results on various objective classes (cores, mitochondria, synaptic vesicles, and film). Due to the scarcity of preparation datasets, new techniques for area adaptation calculations have emerged. They do not yet outperform current estimates for mitochondrial division, but the results are promising. Bermudez-Chacon et al. [44] proposed the area-flexible two-stream U-Net. This method uses preparing information from one area with a lot of training information to work on the division in another space with less preparation. They propose a technique based on double U-Net engineering, in which one stream is used for the source area and another for the objective space. The streams are linked to share a portion of the loads. The Y-Net design, proposed in [45], modifies the traditional encoder-decoder format with an additional remaking decoder to adjust the source and target encoder highlights. They tested their findings by transferring data from isotropic FIB-SEM to anisotropic TEM volumes and cerebrum EM images to HeLa cells.

There are few datasets on the mitochondrial division that are freely accessible to the public. The most commonly used datasets were created by Lucchi et al. [46] and Xiao et al. [47]. According to assessments on Lucchi's dataset, the best methodology for similar creators is the super voxel-based technique [48]. A nonlinear RBF-SVM classifier segmented mitochondria in 3D and 2D data. It is a unique approach that does not rely on CNNs. According to evaluations on the Xiao dataset, the DL approach, which uses 3D spatial data and is proposed by similar creators, is the best methodology. As a variation, they used a 3D U-Net with leftover squares. To address the issue of evaporating inclinations during preparation, they infused assistant classifier layers into the hidden layers.

The segmentation of mitochondria was also attended to for fluorescence microscopy data, where the objective designs were labeled with the use of fluorescence differentiating. The most recent advances are presented, where iterative DL work processes consider the age of beginning great three-layered divisions, which are then used as explanations for preparing DL models. We discovered that no single article segmented the drug, diseased, and mitochondria cell movement-image datasets in related works. We used a new dataset publicly available on Github, and our new model, which is based on CNN, achieved good results. Our model can be used for drug and diseased cell image segmentation on any cell image dataset.

## 3. Method and Explanation

### 3.1. Drug Image Segmentation

Image segmentation is applicable in many scenarios, i.e., content-based image retrieval, machine vision, medical imaging, object recognition, and many other machine vision applications. This study proposed a fully automated image segmentation method for drugs and diseases, which detects highly drug-treated and diseased cell parts from the whole-cell image. The accuracy of new deep learning algorithms (i.e., ResNet-50) relies on extensive datasets for better prediction. Limited datasets have always remained one of the main constraints in medical imaging. To overcome the shortcomings, this study intended to apply different deep learning methods (ResNet-50 and ResNet-152) and finds that ResNet-50 has good performance. Usually, in-depth learning needs a lot of data to be well generalized and overcome the problem of overfitting. A three-dimensional network becomes complex, especially in the case of limited data for training and testing. In the case of two-dimensional activity training, it has some advantages, such as low memory consumption, fast speed, pretrained network, and fine-tuning. In our segmentation method, we trained the model on two-dimensional slices and processed each (drug, normal, and disease) slice independently in training, testing, and validation.

In this article, we used three types of datasets in the form of images (drug-treated image, diseased image, and mitochondrial image). We fed these images to CNN to find segmentation. We compared existing segmentation methods to our proposed method. Our deep learning method is based on Caffe [49], and it consists of two steps: highly treated drug, diseased, and normal detection [50–52] from whole image data. Second, acceptable drug, diseased, and normal segmentation from ROI (region of interest) is localised. The DNDIS (ResNet-50) network's detection part provides ROI and candidates containing the drug. In this method, we assume that contextual information is essential to obtain accurate drug segmentation; in the segmentation part DNDIS (ResNet-50) method, ROI plays a vital role to achieve better segmentation results. This enables the segmentation network to distribute the background pixels of drugs, diseased, and normal cells more evenly and improves versatility and accuracy. In addition, by reducing the size and number of two-dimensional slices introduced into the partitioning network, a more effective partitioning model is obtained.

**Figure 2:**
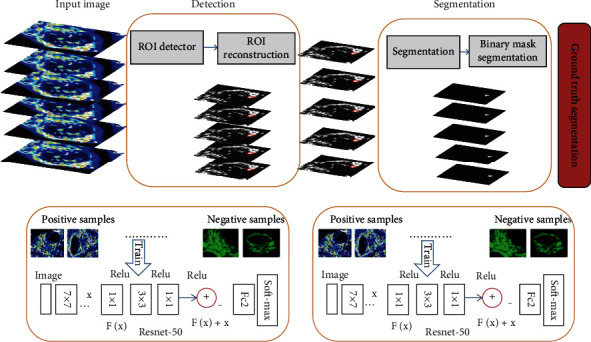
The proposed method for drug, normal, and diseased image segmentation (DNDIS); as an input, drug-treated and diseased cell images; in the second stage, detect that part of highly treated drug and highly diseased cells (ROI) by using ResNet-50 and reconstruct. In the third step, segmentation is identified (binary mask) using ResNet-50; find the ground truth segmented part of drug and diseased part.

In the segmentation stage, the proposed DNDIS outputs a two-dimensional probability map and then processes it to ensure consistency between consecutive slices to achieve the final three-dimensional binary segmentation. A simple and automatic postprocessing step based on three-dimensional *K*-means is adopted in the output probability map to overcome the limitation of two-dimensional approximation and obtain a coherent and accurate three-dimensional binary segmentation mask (see Figure 2). The pipeline construction scheme is introduced, in cases, detection, and segmentation. We used the ResNet-50. In the detection part, our method detects the ROI (region of interest) and then applies the ResNet-50 and finds true positive and true negative caught drug and diseased cell part. We applied the binary mask on the seen part in segmentation parts and used ResNet-50 to find the positive and negative samples and find the correct amount of positive samples (drug and diseased cell) by using the final counter (see Figure 2). Figure 2 shows the architecture of our proposed DNDIS. The hyperparameters for the model, learning rate 0.1, n-neuron 215, iterations 100, N estimator, 1200, and study rate 0.01 were used. The decision function is defined as the objective function of cell image segmentation:

(1) $$\begin{array}{c} S=E\left[S\left|I\right.\right]=\int \text{SP}\left(S\left|I,D\right.\right)\;ds. \end{array}$$

The cross-entropy (*Er*), shape regularisation loss (*Lh*), and weight decay terms are as follows:

(2) $$\begin{array}{c} L_{h}={\left\|f\left(\emptyset \left(r\right);\theta_{f}-f\left(y;\theta_{f}\right)\right.\right\|}_{2}^{2},\\ \min_{\theta }\left(E_{r}\left(\emptyset \left(r;\theta \right)-y\right)+{∆}_{1}L_{h}+\frac{{∆}_{2}}{h}\right){\left\|W\right\|}_{2}^{2}. \end{array}$$

As in, the decision for class labels is computed using pixel-wise softmax.

(3) $$\begin{array}{c} \mu =\left(\frac{1}{M}\right)\overset{M}{\underset{k=1}{\sum }}x_{k},\\ x_{k}^{\prime }=x_{k}-\mu ,\\ \sum =E\left(xx^{T}\right)=\frac{1}{M}\overset{M}{\underset{k=1}{\sum }}\left(x_{k}^{\prime }\right){\left(x_{k}^{\prime }\right)}^{T},\\ x_{n}^{l}=f\left(\underset{m}{\sum }x_{m}^{l-1}*W_{mn}^{l}+b_{n}^{l}\right). \end{array}$$

### 3.2. Using Deep Detection Network to Detect Drug, Diseased, and Mitochondrial Cell Part

**Figure 3:**
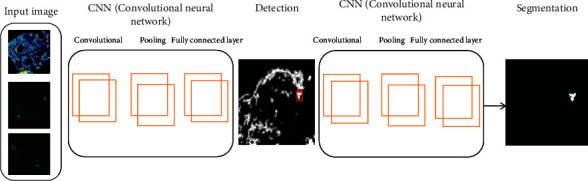
Deep detection and segmentation model based on the ResNet-50.

Deep detection is the core component of our DNDIS (drug, normal, and diseased image segmentation) system. Figure 2 illustrates the architecture of DNDIS. In the deep detection method, the convolutional features are mapped, and the reference box is called anchor; the siblings are responsible for classifying regressing bounding box and anchor. Two main things are done; the first is the anchor box, which predicts the foreground object using an anchor classifier, and the bounding box estimates the thing's location. Secondly, using the predicted coordinates, the anchor's box transforms to the region proposed for each object, mapping features through the region of interest (ROI), pooling layer extracts a fixed-length quality, and feature vector feeds the part classification network. The network has a sibling output layer, the softmax layer and encoding bounding boxes. We sample the image patches; the 16-pixel convolutional feature map is equivalent to 8 pixels in the original image. The DNDIS used three anchors with areas of (128 2, 256 2, and 512 2) pixels for different objects detection (see Figure 3). In Figure 3, the first part of our method is the detection by using ResNet-50.

This deep detection and segmentation model is based on the ResNet-50; in the first step, different types of cell images are taken as input, and here, we illustrate the building blocks in the second step where we used ResNet-50 to detect the highly treated drug part from cell images. The red box is the last detection image mark, false positive, and genuinely positive. And finally, identified the segmentation of the highly treated drug image part.

Drug and diseased detection task is an object detection problem, so the proposed method is diseased, normal, and drug image segmentation (DNDIS) by using ResNet-50 to solve this problem; to the best of our knowledge, this is the first study that applies deep learning, to detect and segment drug-treated cell image problem. Redmon et al. [53] proposed the region-based detection model. We adopted the model to see the drug and diseased detection task. Our proposed method of deep segmentation is illustrated in Figures 2 and 3. Our approach has three main components: the first is the deep segmentation method that produces the estimated bounding box (see Figures 2 and 3). Secondly, the localising drug and diseased part and ResNet-50 use deep classification detection patches to improve accuracy. For training of the deep detection model, it demands the bounding box labels. We can train the detector on drug and diseased datasets and give each pixel a title.

For the centre of mass of each tag, we infer the boundary box by combining the segmentation result and the centre of mass identification. Then, the depth detector is trained by using the prediction box label (Figure 3). Firstly, we run a depth detection model on mitochondrial images to generate detection results and then input these detected image patches into the depth verification model for further refinement. The verification model is a ResNet-50, a robust classification network [54]. Finally, the weighted sum of the detection model and verification model prediction was obtained.

**Figure 4:**
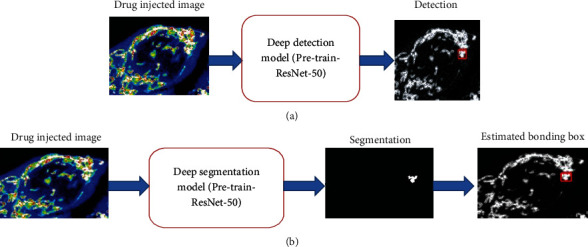
(a) Drug, normal, and diseased detection system overview (DNDD), the deep segmentation, drug injected image, and second step used deep segmentation model, using ResNet-50. And the third step finds the true positive and segmentation part. The fourth step is the estimated bounding box. (b) See the segmentation, highly drug-treated or diseased detail, and verification.

In short, this paper has at least four main contributions. Firstly, we have obtained the latest results of three challenging drug, normal, and diseased detection datasets, which are fast (see Figure 4(a)), and secondly a universal target detection framework for medicine and diseased detection (see Figure 4(a)). As far as we know, this is the first time that CNN has been applied in depth detection technology to drug and diseased detection. Thirdly, a training scheme for weak monitoring of boundary box detectors has been proposed. The boundary box label is estimated by depth segmentation network (see Figure 4(b)). This soft supervised detector learning method can significantly reduce the labeling work of pathologists. Fourthly, the classification model verifies the detection results further (see Figure 4(b)). The deep validation model provides a bootstrapping mechanism for mining hard and negative samples. Combining the depth detection model with the depth verification model can improve the system's performance (see Figures 4(a) and 4(b)). Figures 4(a) and 4(b) show the architectural diagram of drug, normal, and diseased detection system overview (DNDD).

## 4. Experimental Results

This section evaluates the performance of the proposed method: drug, normal, and diseased image detection (DNDIS) on drugs, standard, and diseased image datasets. The whole system deep learning drug segmentation (DLDS) is implemented based on the Caffe using python [49].

### 4.1. Dataset

**Figure 5:**
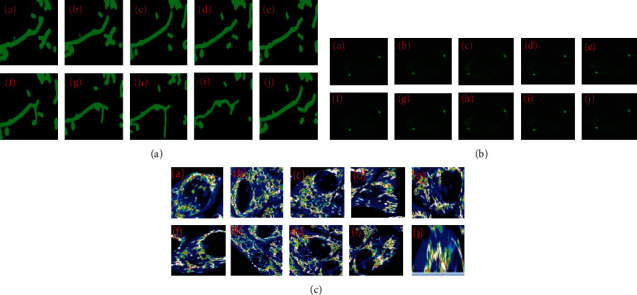
Datasets which were used for experiments: (a) normal mitochondrial cell images, (b) diseased cell images, and (c) drug-treated images.

Images of drug, typical, and diseased cells were captured by confocal microscopy. Ten pictures of normal cells images are shown in Figure 5(a). Photos from 1 to 10 show minor variations in the position of normal cells. Figure 5(a) shows normal cells that got damaged after some time, normal cell apoptosis, and renewable cells. As shown in Figure 5(b), ten pictures of diseased cells, which show no variations, cell growth is continuous without any sign of apoptosis or replacement of old cells by new healthy cells. Ten pictures of drug-treated cells images are shown in Figure 5(c). Photos from 1 to 10 show minor variations in normal cells position.  Table 1 shows a detailed description of the dataset used for the experiments. Additionally, it also represented the specifics of each type of image. We evaluate our method on microscopy image, resolution of 0.2456 *μ*m per pixel, and image area is 512 × 512 *μ*m and size of image 2084 × 2084 pixels [55–57].

### 4.2. Deep Detection Method Drug, Normal, and Diseased Detection Dataset

On the basis of the ground truth of drug, diseased, and normal cell dataset, through the DNDIS method, we can quickly obtain an accurate bounding box for the training of our model. The model can yield excellent performance, and we do not need a verification model on this dataset.

### 4.3. Drug, Diseased, and Normal Cell Region of Interest Detection

The proposed method DNDIS, ROI (region of interest) detector, is based on the deep architecture; the main aim of this method is to detect the absence or presence of the drug, diseased, and normal cell in every slice of three different types of data. To determine the bounding box around the three other images and get the region of interest, generate the area of interest of upcoming segmentation. The two main objectives are as follows: firstly, detect the initial and final slices (*A*_min_ and *A*_max_), where the drug, diseased, and normal cells are visible from the whole dataset and the secondly, determine the rectangular region around the drug, diseased, and normal cells in each slice. From a medical point of view, when the cell wall expends more than 6 mm, the presence of the drug and diseased cells can be assumed. Through visual inspection, approximately the region of interest will be determined in practice, and it has two observers; intra- and interobserver variability exists. Regarding the results of the normal, diseased, and drug cells in each slice of the dataset, we have selected three different experts, the first observer manually indicated the final and initial drug and diseased slice, and it depends on the observer's judgment. So the interobserver variability (IOV) is measured for the last and initial portions of the drug and diseased cells. One main aim is the selection of pieces, and it is taken based on the standard deviation of three observers.

Our interest depends on choosing a reduced area from the whole drug, standard, and disease image dataset, including medicines and diseases, even with some adjacent sections [58]. Therefore, we focus on minimising the false-negative rate (FNR), i.e., the ratio of undetected drugs and normal and diseased slices to total drug, normal, and diseased slices, taking into account the average of all previous expert observers. The average FNR of all datasets and networks is 0.086 + 0.107. The results are summarized in Table 2. The maximum false-negative rates for M1 and M3 are due to datasets with special characteristics: one is a vast drug that is diseased compared to the average size, which also affects segmentation and will be explained later; the other is that drugs and diseases extend to the iliac artery and discards it because it is not considered in training.

**Figure 6:**
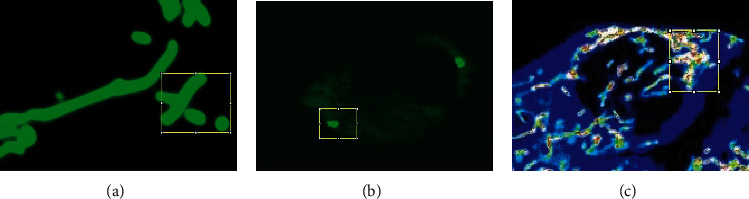
Example of some detected bounding boxes in the three datasets (normal mitochondrial, diseased, and drug). (a) is the normal mitochondrial cell image bonding box, (b) is the diseased cell image bonding box, and (c) is the drug-treated cell image bonding box.

The minimum and maximum *X* and *Y* coordinates of all boundary boxes are selected for the two-dimensional boundary boxes that divide the drug, normal, and diseased cells in each slice. Then, we expand the area, including broader context information necessary for good segmentation. Therefore, we always get a three-dimensional region of interest, which can correctly define the drug and diseased in *X* and *Y* in all cases. Some visualization examples are given in Figure 6.

### 4.4. Drug-Treated and Diseased Cell Image Segmentation

**Figure 7:**
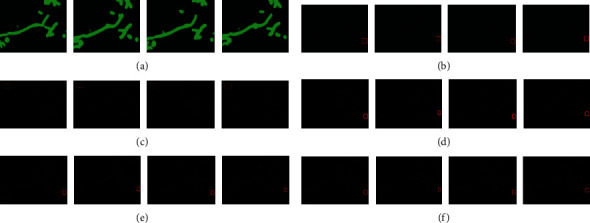
Dataset of normal mitochondrial cell images. In this figure, the dataset of normal mitochondrial cell images, we just take four image patches and normal mitochondrial image slices. We compared our method with nondeep learning methods. Segmentation results of the real dataset. (a) Original image, (b) ground truth, (c) DRLSE, (d) proposed method, (e) SSDMT, and (f) LACM-BIC.

**Figure 8:**
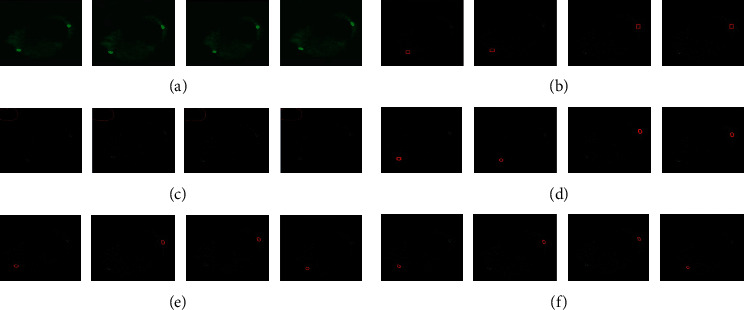
Dataset of diseased cell images. In this figure, the dataset of diseased cell images, we just take six image patches and diseased image slices. We compared our method with nondeep learning methods. The segmentation results of the real diseased dataset. (a) Original image, (b) ground truth, (c) DRLSE, (d) proposed method, (e) SSDMT, and (f) LACM-BIC.

**Figure 9:**
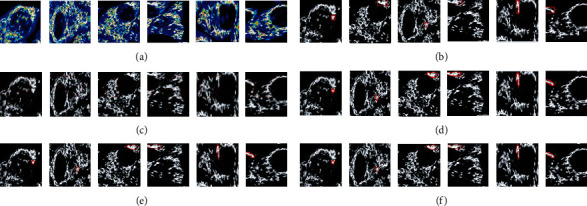
Dataset of drug-treated cell images. In this figure, we just take six image patches and drug-treated image slices. We compared our method with nondeep learning methods. The segmentation results of the real drug dataset. (a) Original image, (b) ground truth, (c) DRLSE, (d) proposed method, (e) SSDMT, and (f) LACM-BIC.

**Figure 10:**
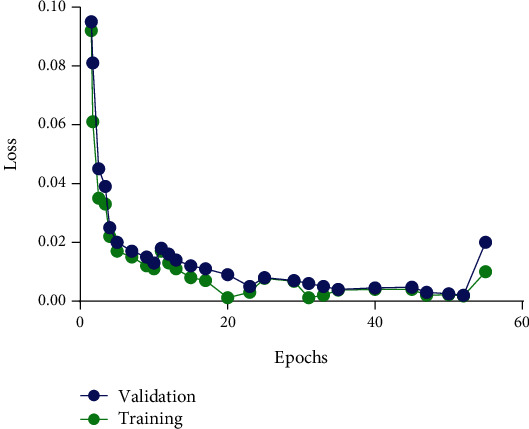
Training and validation loss of CNN method. The Adam algorithm minimised weighted cross-entropy loss; the *x*-axis shows the number of epochs and the *y*-axis is lost.

Here, we present the results of our method and detect the edges and preserve the appearance of drug and diseased cells. For comparison of our method with other methods, our methods has indeed improved the segmentation accuracy. And it is beneficial for the drug and diseased cells. In this article, we have fine-tuned and tested the network. Our method is compared to the results with the other two approaches (see result section). We trained and tried our method in the method section and got good segmentation accuracy (see Table 2). We used three different datasets (routine mitochondrial [44, 45] cell, diseased, and drug). And we compared our method with other two different methods (SSDMT and LACM-BIC), and the result of segmentation is presented in Figures 7–9 and Table 2. First, in our approach, the learning parameters (biases and weight) are reduced, the training and validation losses decreased, and testing and training times are also lesser than other methods. One more thing in our proposed method is fine-tuning and finding the better results of improved segmentation of the normal, diseased, and drug cells. Table 2 compares deep verses nondeep learning segmentation in deep learning; network training and validation are shown in Figure 10. We compared different methods and reached our method (DNDIS) with some other methods; Table 2 and Figure 7–9 show the details. Our method achieves high accuracy on drug data. As a result, our deep learning-based method gives better results than other baseline methods. Our method gives excellent results on the drug-treated images (see Figure 9).

### 4.5. Model Validation in terms of Performance

The high drug-treated count is critical in medical industries; when medical organisations produce some new medicines, first there is a need to test the effect of newly created treatment, essential to measure how much it can damage the normal cell, so the measurement of performance in high drug-treated detection task is based on the number of correctly detected high drug-treated cells, rather than the shape of detected high drug-treated cells. Similarly, in the diseased, for identification of diseased (clinical medicine, radiology, pathology, and cancer) [59–61], also measurement of performance is based on the diseased detection task, and correct detection of diseased cells, not the shape. The correct detection criteria of drug and diseased cells are a distance from the centroid of ground truth drug and diseased cells. In this article, we defined some measures of drug, diseased, mitochondrial, and normal cell accuracy. True-positive (TP) cells are those that have been exposed to a drug, are diseased, have mitochondria, or are normal. In contrast, false positive (FP) is detecting positive not ground truth drug and diseased cells, and undetected drug and diseased cells are false negative (FN). According to these measures, the false-positive rate is calculated using the equation FP/FP + TN, where FP represents false positives. TN represents true negatives (FP + TN = total number of negatives). It is the likelihood of a deception being set off, with a positive outcome when the true worth is negative; we can calculate the precision, recall, and *f*-score [35, 36, 62] using the following:

(4) $$\begin{array}{c} \text{Recall}=\frac{\text{True Positive }}{\text{True Positive}+\text{False Positive}},\\ \text{Precision}=\frac{\text{True Positive }}{\text{True Positive}+\text{False Positive}},\\ F-\text{score}=\frac{2\times \text{Recall}\times \text{Precision }}{\text{Recall}+\text{Precision}},\\ \frac{\text{False Positive }}{\text{Total Number of Negative}}=\frac{\text{False Positive }}{\text{False Positive}+\text{True Negative }}. \end{array}$$

## 5. Discussion

Image segmentation is the method to partition the image into multiple segments; the main objective of segmentation is to analyze meaningful image representation. In medical imaging segmentation, the small image segments correspond to different tissue (organs, classes, pathologies, normal, drug, and diseased cells) or other biologically related structures. In this article, we have proposed a method DNDIS (drug, normal and diseased images segmentation) that fully automated segmentation of the drug and diseased cells. Our method first detects the drug and diseased cell region from the whole dataset. In this method, there is no need for user interaction at any stage, and there is no prior knowledge for the shape detection drug and diseased cells, because our process is fully automated. In our method, the following hyperparameters are used for training the network CNN (convolutional neural network), weights (Xavier) and Bias (0.10), input image size 2D (512 × 512 × 3), optimiser (Adam), batch size (1), and learning rate (5e^5^).

In some previous studies, automated segmentation techniques were evaluated in authors who proposed mitotic cell detection, multiregion image segmentation, greenhouse image segmentation, MR image segmentation, brain [63, 64] image segmentation, and nuclear image segmentation [40]. In the previous methods, some traditional machine learning and deep learning methods were applied for image segmentation in different fields like a tumour, MRI. Despite this, no method for drug-treated image cell segmentation was found in the literature. The authors propose a method for segmenting drug-treated images, as well as diseased and normal cells. We have compared our method with some previous traditional methods and found that the accuracy of our method is better than other methods; especially, our method gives excellent results on drug-treated image datasets. We hope our method will be beneficial to drug-manufacturing companies; they can check the new drug effect on normal cells through segmentation. Because drug testing is the main problem in the modern age, sometimes medical companies use animals to test their newly made drug. We believe that our method is a small contribution to measuring the drug effect. Figure 10 shows the training process of our proposed method.

**Figure 11:**
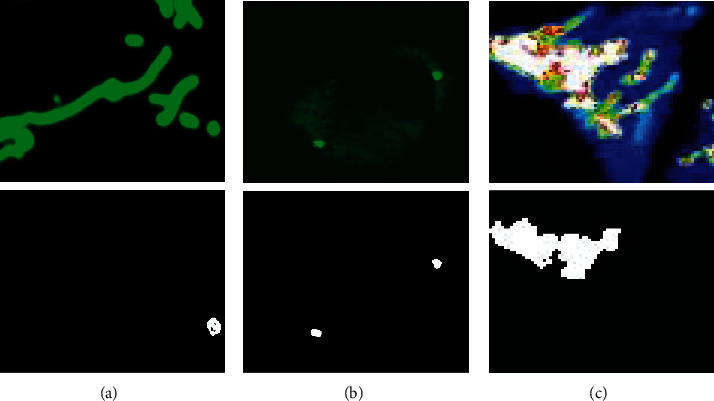
Our own proposed method (DNDIS) results (high drug-treated and diseased part). (a) shows the segmentation of normal mitochondrial cell image, (b) shows the diseased cell image segmentation, and (c) shows the drug-treated cell image segmentation.

We compare two effective methods: SSDMT (single seed delineation with multithresholding) and LACM-BIC (localized active contour model with background intensity compensation), and DRLSE was used for medical image for both methods and used pixel-level image segmentation. Our experiments explain that through CNN (ResNet-50), to detect the highly drug-treated and diseased cell image patches and improve the performance, in the second phase, segmentation is done and gives true positive as a result (segmentation). The automated segmentation using ResNet-50 has the potential to bring the use of the newly made drug for any medicine company and cell diseased part measurements into the clinical practice. Some results of our method are shown in Figure 11.

## 6. Conclusion

The first analysis of cells using computer technology dates back to fifty years. Now, image segmentation is primarily used in cell imaging and is considered a substratum in image analysis. Many machine learning techniques have been proposed for analyzing image data, but deep learning methods shepherd all state-of-the-art techniques. This article proposed the drug, normal, and diseased image segmentation (DNDIS) method for highly drug-treated part segmentation in slide images. We adopted some general methods of segmentation (i.e., SSDMT and LACM-BIC) and achieved excellent performance on the drug image dataset. The datasets of drug and diseased cells do not provide acceptable ground truth (bounding box); we exploit a segmentation method to estimate the drug and diseased regions. The experimental results revealed that our segmentation method is better than traditional methods and improves the detector's performance. The method's effectiveness is demonstrated by reducing the time spent manually labeling images in a medical image analysis system. Compared to other techniques, this method outperforms image data, and this image segmentation technique can be applied to any image dataset.

The proposed method for segmenting mitochondrial, drug, and diseased cell images has been used in medicine testing (newly developed drug), clinical medicine (cancer and clinical pathology), and oxidative stress. Our method is useful for drug testing, to test newly developed drugs; in diseased cells, the proposed method is very useful to segment those parts of image that are diseased; it will be useful in medical fields such as cancer detection and imaging; and finally, our method is useful for mitochondrial cell image segmentation, which is useful for measuring oxidative stress. This task can be improved further by creating new models for mitochondrial, diseased, and drug-treated image segmentation, cell image localization, drug effect on cell imaging, drug testing via deep learning and mitochondrial cell segmentation applications, measuring oxidative stress more accurately, and finally creating a new dataset for experiments.

## Figures and Tables

**Table 1:** Description of the dataset.

Image types	Number of images	Training	Test
Drug treated [55]	138	100	38
Diseased [57]	38	30	8
Mitochondrial cell movement [56]	142	100	42

**Table 2:** Results obtained from different methods on the drug-treated dataset. Accuracy of different methods over mitochondrial cell images slice of 10 cell types.

Cell type	SSDMT			LACM-BIC			Proposed (DNDIS)		
#	Normal	Diseased	Drug	Normal	Diseased	Drug	Normal	Diseased	Drug
1	0.8092	0.8124	0.8536	0.8123	0.8233	0.8669	0.8559	0.8799	0.9533
2	0.8211	0.8422	0.8688	0.8567	0.8744	0.9011	0.8663	0.9057	0.9611
3	0.8122	0.8249	0.8465	0.8259	0.8438	0.9133	0.8547	0.8956	0.9025
4	0.7898	0.8129	0.8564	0.8012	0.8268	0.8561	0.8186	0.8519	0.9023
5	0.8521	0.8700	0.8845	0.8701	0.8897	0.9087	0.8894	0.8963	0.9469
6	0.8402	0.8630	0.8940	0.8599	0.8699	0.9123	0.9054	0.9146	0.9512
7	0.8356	0.8591	0.8816	0.8451	0.9036	0.9122	0.9011	0.9259	0.9615
8	0.8559	0.9025	0.9175	0.8644	0.9156	0.9328	0.8945	0.9265	0.9628
9	0.7999	0.8413	0.8527	0.8319	0.8524	0.8898	0.8749	0.9129	0.9314
10	0.8683	0.8871	0.9023	0.8721	0.8936	0.9082	0.9091	0.9283	0.9473

## Data Availability

Dataset link is available in the article; dataset is available publicly.

## Abbreviations

MCI: Mitochondrial cell imaging
DNDIS: Drug-treated, normal, and diseased image segmentation
CNN: Convolutional neural network
MRI: Magnetic resonance imaging
ResNet-50: Residual networks-50
ResNet-152: Residual networks-152
ROI: Region of interest
DNDD: Drug, normal, and diseased detection
FNR: False-negative rate
IOV: Inter observer variability
TP: True positive
FP: False positive
FN: False negative
SSDMT: Single seed delineation with multithresholding
LACM-BIC: Localized active contour model with background intensity compensation.

## Supplementary Materials

Supplementary Materials The supplementary files contain the results Comparison of different models, Data type Image and single image result segmentation, and Model Training figures.
