# Supplementary material for: Segmentation of Drug-Treated Cell Image and Mitochondrial-Oxidative Stress Using Deep Convolutional Neural Network
Source: Oxid Med Cell Longev. 2022 May 26;2022:5641727. doi: 10.1155/2022/5641727 (PMC9162846; doi:10.1155/2022/5641727)
Supplement: Supplementary Materials — The supplementary files contain the results Comparison of different models, Data type Image and single image result segmentation, and Model Training figures. [file 5641727.f1.docx]

**Results Comparison of different models**


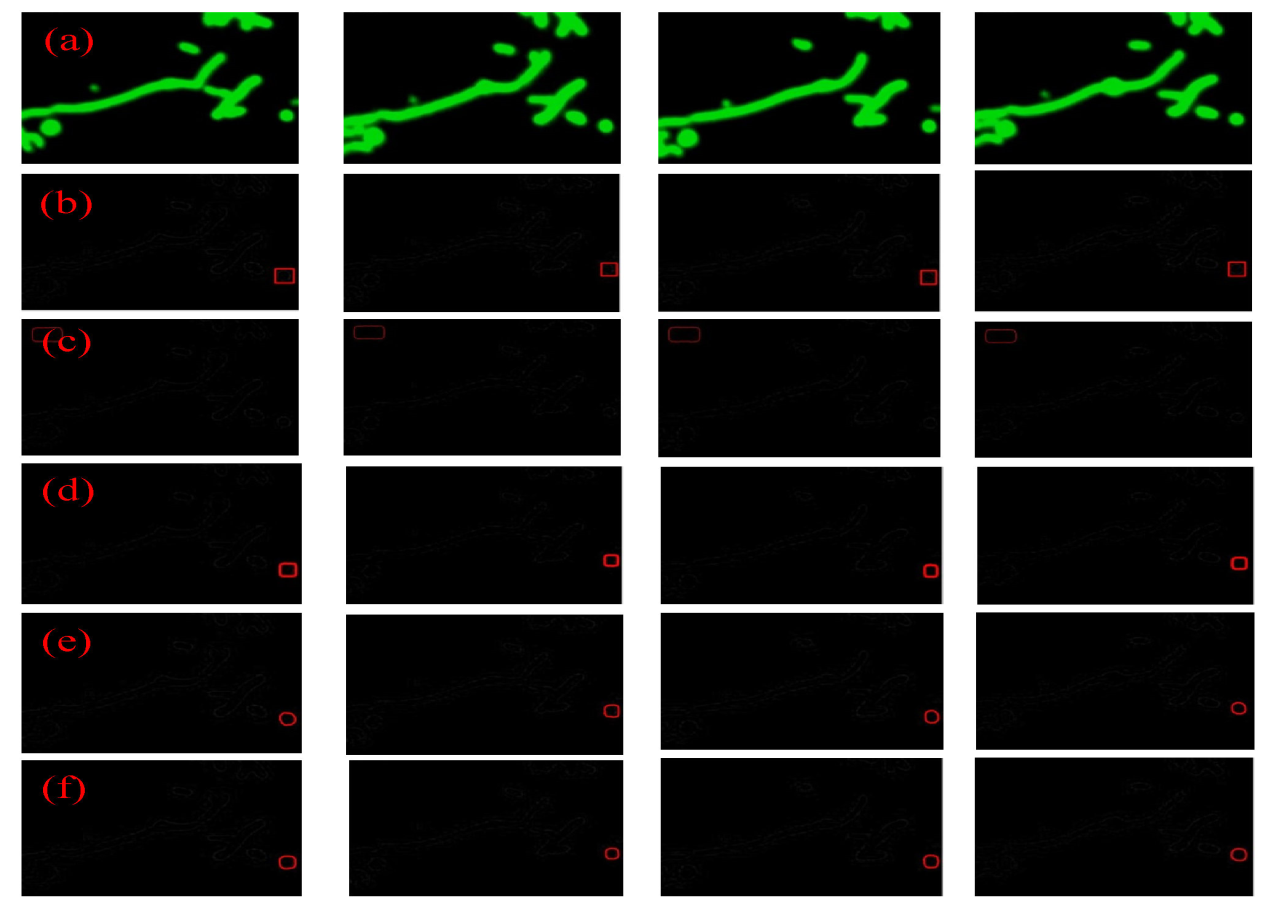


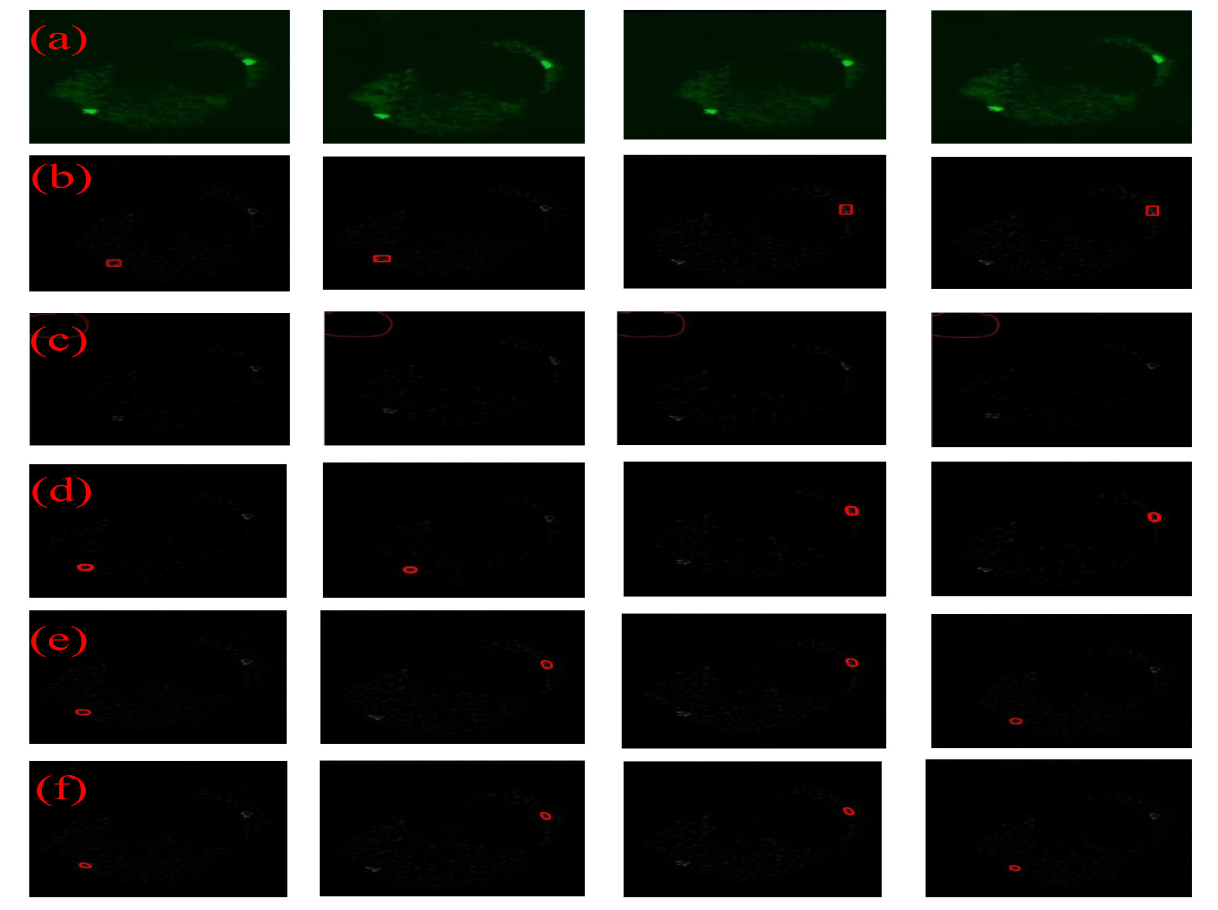


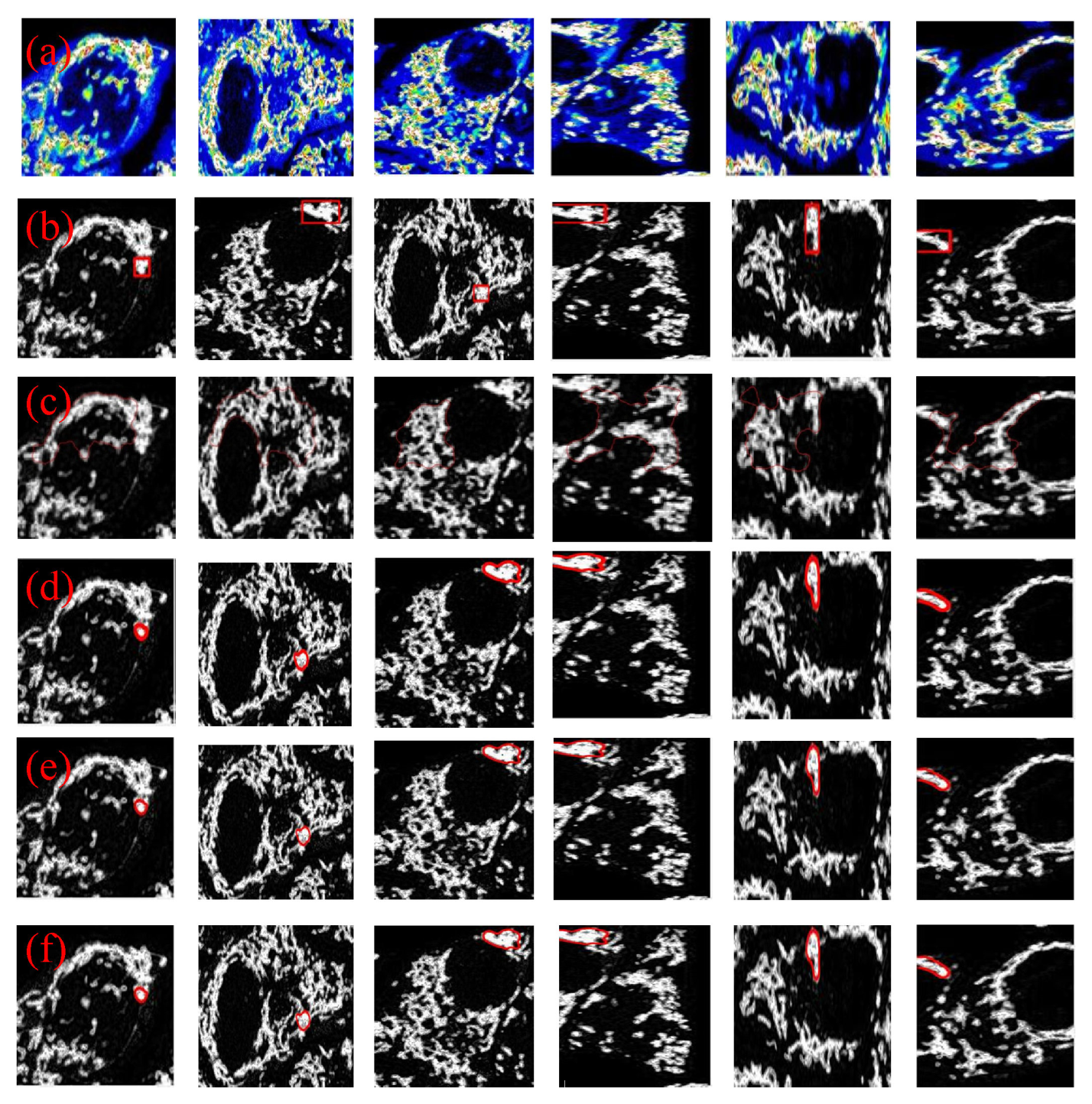


**Figure (a)**. In this figure the dataset of normal mitochondrial cell images, here we just take four image patches, normal mitochondrial image slices. We compared our method with non-deep learning methods. Segmentation results of real dataset. (a) Original image, (b) ground truth,(c) DRLSE,(d) proposed method, (e) SSDMT and (f) LACM-BIC. **Figure (b).** In this figure, the dataset of diseased cell images, here we just take six image patches, diseased image slices. We compared our method with non-deep learning methods. The segmentation results of real diseased dataset. (a) Original image, (b) ground truth, (c) DRLSE, (d) proposed method, (e) SSDMT and (f) LACM-BIC. **Figure (c).** In this figure, the dataset of drug treated cell images, here we just take six image patches, and drug treated image slices. We compared our method with non-deep learning methods. The segmentation results of real drug dataset. (a) Original image, (b) ground truth, (c) DRLSE, (d) proposed method, (e) SSDMT and (f) LACM-BIC

**Data type Image and single image result segmentation**

**
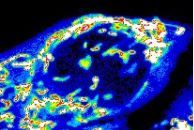

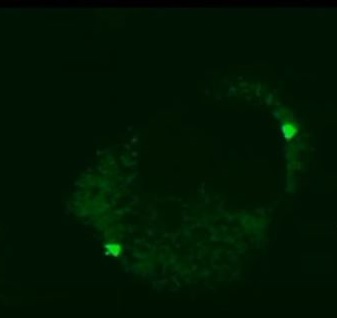

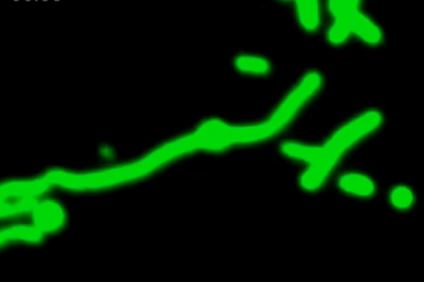
**

**
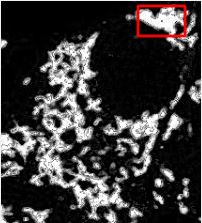

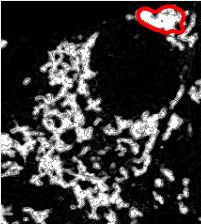

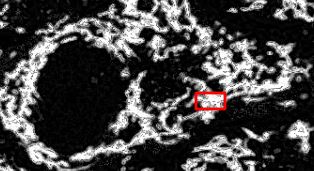
**

**
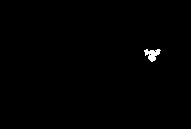
**

**Model Training**

**
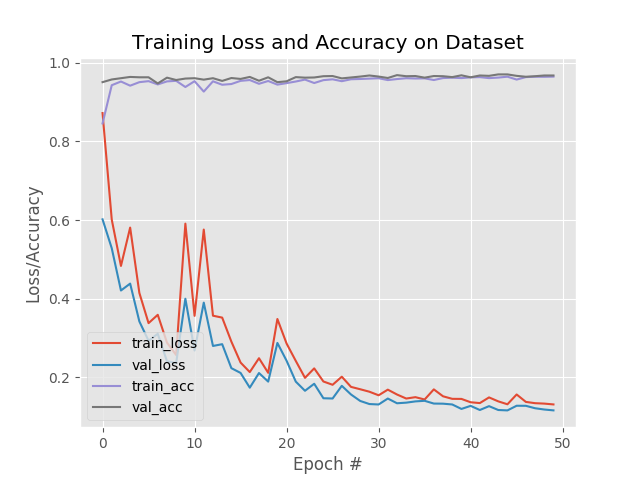
**
